# Diffuse large B-cell lymphoma originating from the rectum and diagnosed after rectal perforation during the treatment of ulcerative colitis: a case report

**DOI:** 10.1186/s12893-021-01060-2

**Published:** 2021-01-21

**Authors:** Takafumi Suzuki, Kazutsugu Iwamoto, Ryoichi Nozaki, Yasumitsu Saiki, Masafumi Tanaka, Mitsuko Fukunaga, Kazutaka Yamada

**Affiliations:** 1 Department of Surgery, Coloproctology Center Takano Hospital, 3-2 Ooe, Kumamoto, Kumamoto 862-0971 Japan; 2 Department of Surgery, Shinkuki General Hospital, 418-1 Kamihayami, Kuki, Saitama 346-8530 Japan; 3 Department of Gastroenterology, Coloproctology Center Takano Hospital, 3-2 Ooe, Kumamoto, Kumamoto 862-0971 Japan

**Keywords:** Diffuse large B-cell lymphoma, Ulcerative colitis, Intestinal perforation

## Abstract

**Background:**

Gastrointestinal lymphomas like diffuse large B-cell lymphoma (DLBCL) are rare complications of ulcerative colitis (UC), and only a few studies have reported intestinal ulcers caused by DLBCL, which got perforated during the treatment of UC.

**Case presentation:**

A 43-year-old man with severe lower abdominal pain and an 8-year history of UC was admitted in our hospital. He was diagnosed UC since 8 years and received a maintenance oral dose of 5-aminosalicylic acid, and no other immunosuppressive drugs. A deep rectal ulcer was endoscopically diagnosed 10 months before admission, no malignancy or cytomegalovirus infection was detected on biopsy. After 7 months a further endoscopy with biopsies confirmed the finding and the absence of malignancy. Three months later the patient developed sudden abdominal pain and was admitted in our hospital. Rectal perforation was suspected on X-ray and computed tomography imaging, and an emergency surgery was performed. Surgical exploration revealed a perforation on the anterior wall of the rectum. A subtotal colectomy with temporary ileostomy was performed. Pathology examinations showed lymphocyte infiltration of all of the layers of the perforated site and an immunohistochemical evaluation revealed DLBCL. Clinical staging was stage IV, and the patient received a 6-months regimen of R-CHOP (rituximab, cyclophosphamide, doxorubicin, vincristine, and prednisolone) chemotherapy. Positron emission tomography restaging revealed disappearance of distant uptake and a slight uptake in the residual rectum, and completion proctectomy with ileal pouch-anal anastomosis was performed. No residual tumor in the specimen was found, and the patient was disease-free at 2 years follow-up.

**Conclusions:**

DLBCL may increase the frequency of perforation and is a poor prognostic risk factor for patients with UC. This case study emphasizes the importance of careful medical surveillance and repeated endoscopic biopsies during the treatment of UC.

## Background

Intestinal perforations are rare in patients with ulcerative colitis (UC) since the inflammation is usually limited to the submucosal layer. The incidence of perforation in UC is reported to be approximately 3% [1]. On the other hand, the incidence rate of perforations in gastrointestinal lymphoma is relatively high [2–4]. Therefore, gastrointestinal lymphomas can increase the risk of perforation in patients with UC.

Colon cancer is a common complication of UC, but inflammatory bowel disease (IBD), on its own, is not recognized as a risk factor for gastrointestinal lymphoma [5–8]. Previous studies have shown that long-term immunosuppressive drugs and tumor necrosis factor (TNF) inhibitor therapy in patients with UC induce gastrointestinal lymphoma [9, 10]. It has been suggested that long-term therapy with immunosuppressive drugs and TNF inhibitor cause immunosuppression and increase the incidence of Epstein-Barr virus (EBV)—positive lymphomas (i.e., diffuse large B-cell lymphoma (DLBCL)) [11]. However, other studies have reported cases of DLBCL in UC patients not treated with immunosuppressive drugs and TNF inhibitors [12, 13].

Here, we report a case of rectal DLBCL that got perforated while the patient was being treated with 5-aminosalicylic acid (5-ASA) and prednisolone for UC.

## Case presentation

**Fig. 1:**
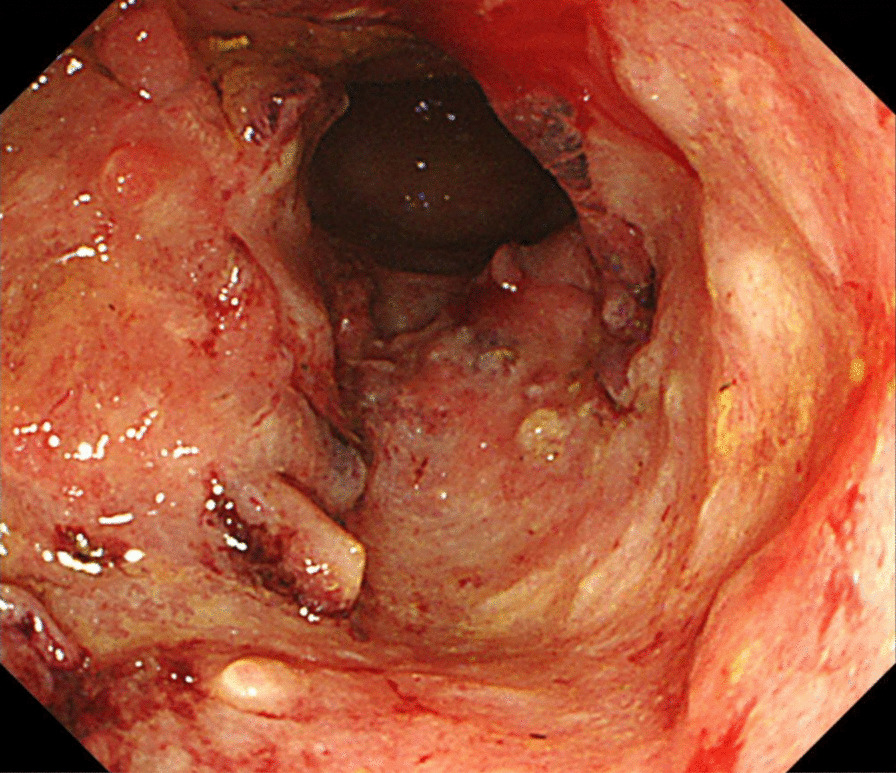
Endoscopy examination. The deep ulcer was detected and diagnosed grade 3 according to the Mayo endoscopic subscore

**Fig. 2:**
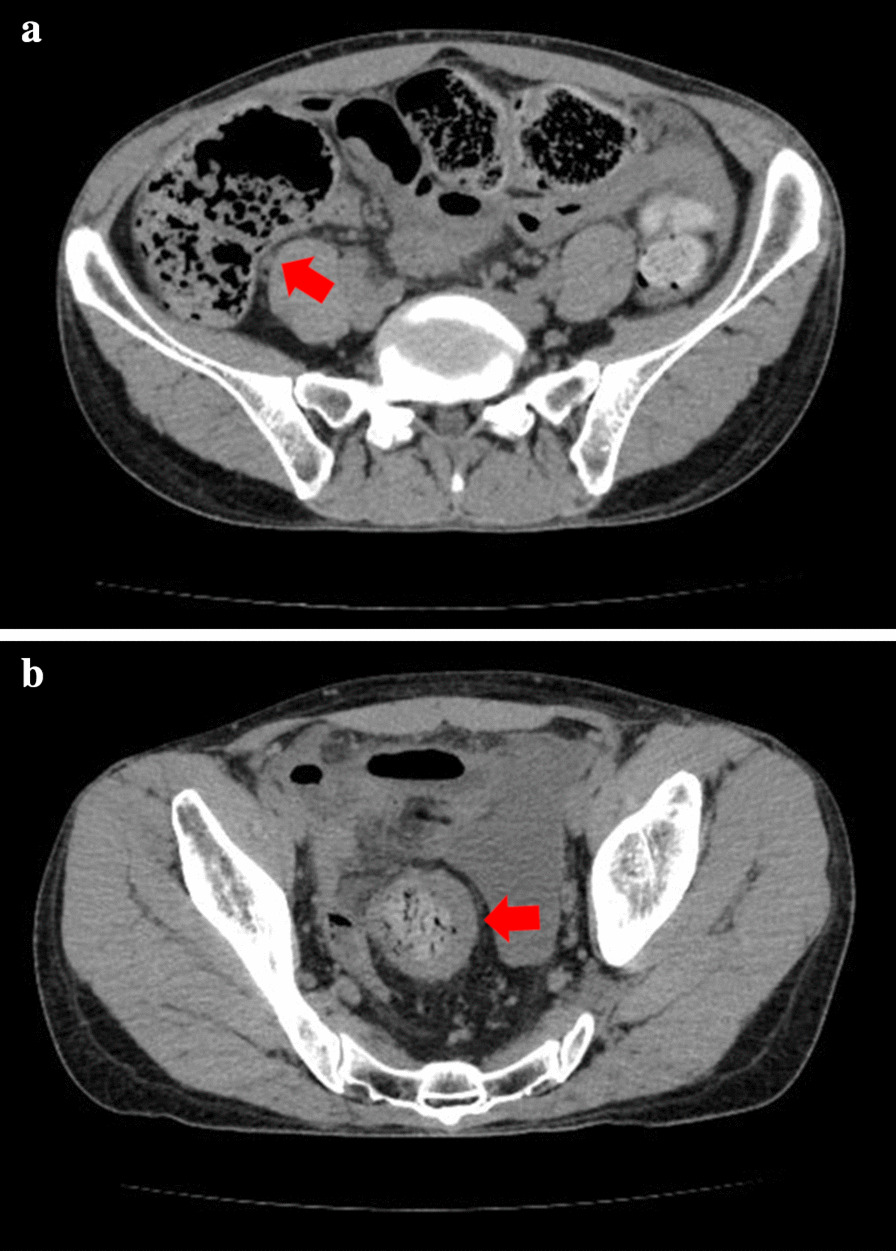
Preoperative CT findings. Abdominal CT scan showing intraperitoneal free air and colon distension, suspicious of gastrointestinal perforation (**a**). Pelvic CT scan showing the perforation site in the rectum but does not clearly reveal the presence of the lymphoma (**b**)

**Fig. 3:**
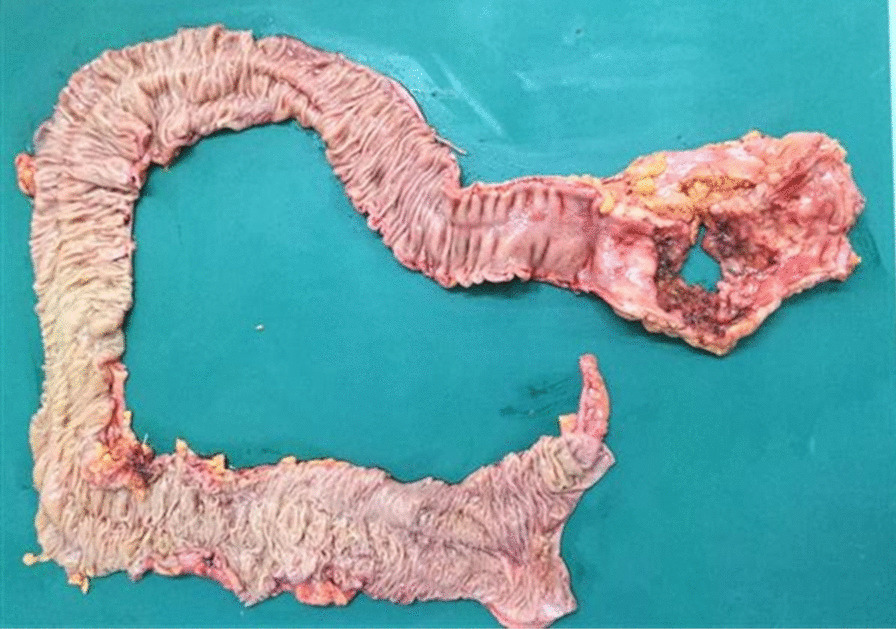
Resected rectosigmoid colon specimen. The perforation site is visible on the resected rectosigmoid colon. The mucosa is mostly normal except for the perforation site

**Fig. 4:**
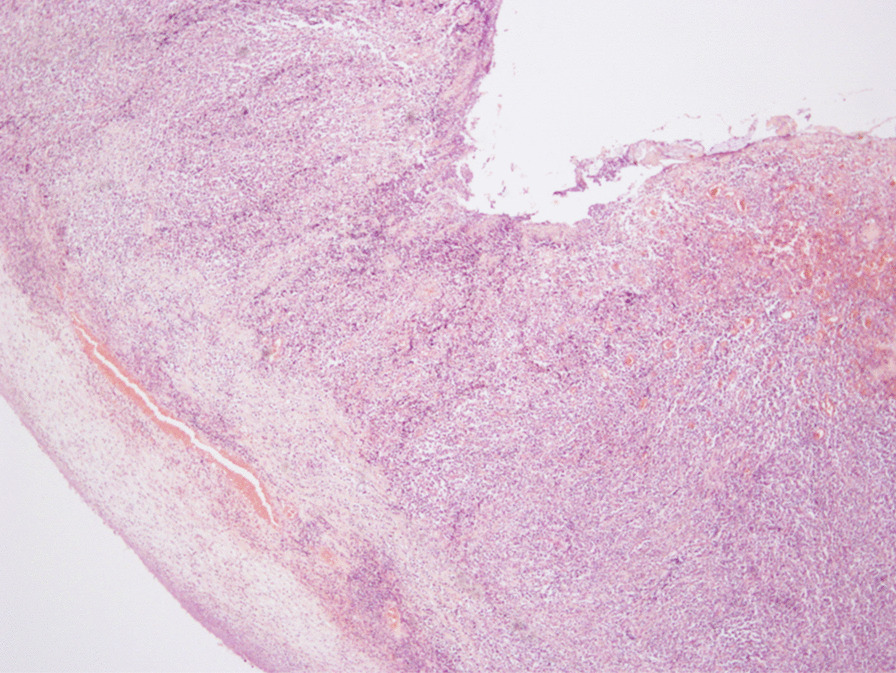
H&E stained slide from the perforation site. Pathological examination of the resected specimen showing atypical lymphocytes infiltration in all the layers of the rectal wall (× 40)

**Fig. 5:**
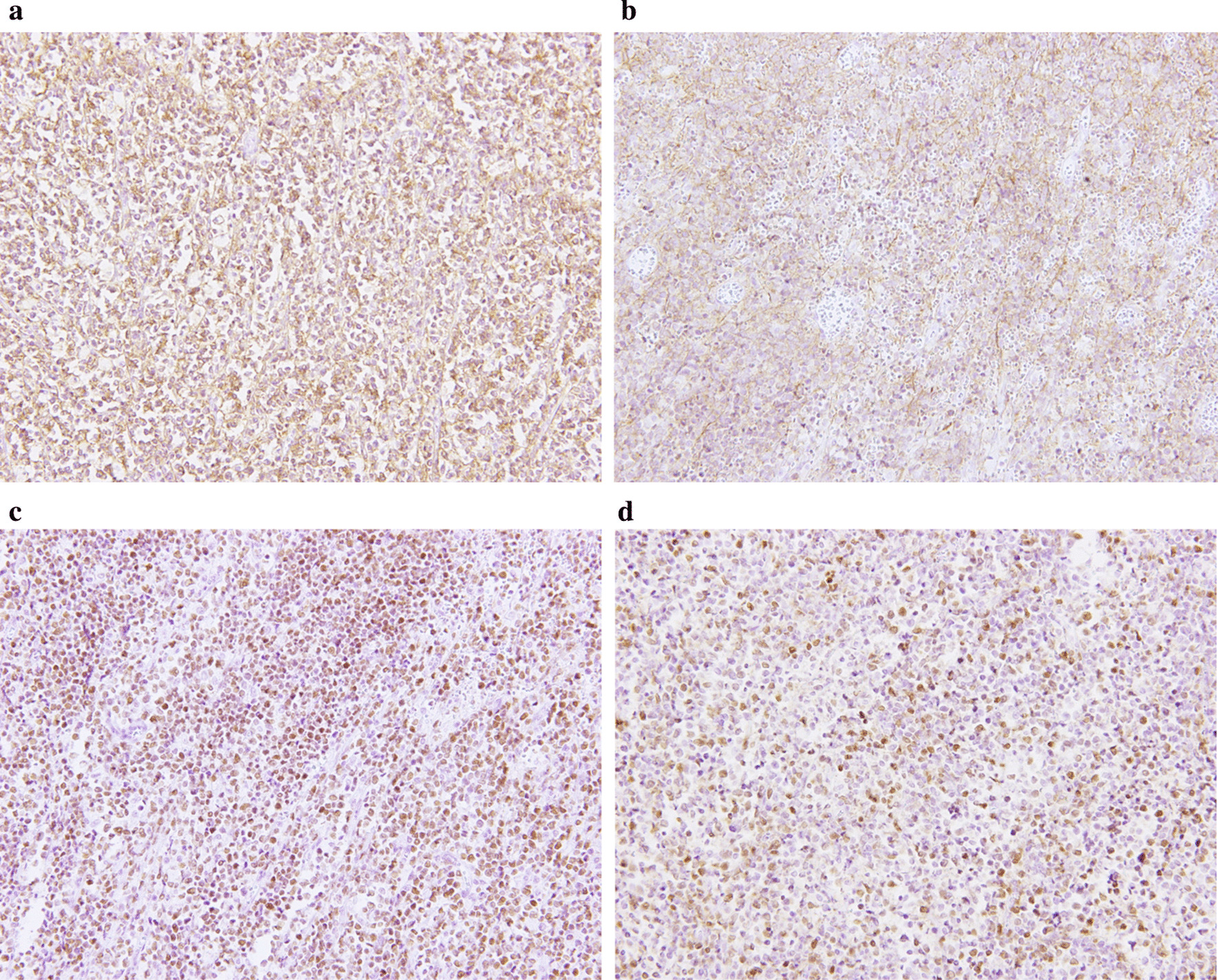
Immunohistochemical examinations from the perforation site. Atypical lymphocytes are CD 20 positive (**a**), CD 10 positive (**b**), BCL-6 positive (**c**), and MUM-1 positive (**d**), (× 200)

**Fig. 6:**
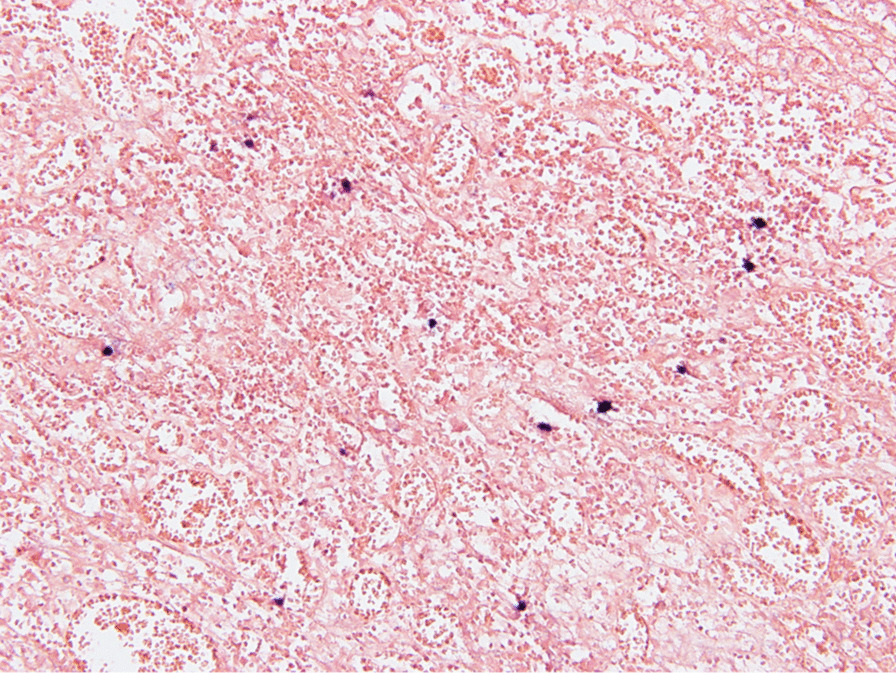
Tumor cells are positive for EBV on in situ hybridization, suggesting that tumor cells are infected with EBV (× 200)

A 43-year-old man with severe lower abdominal pain was admitted to our hospital our hospital for medical treatment. He had an 8-year history of ulcerative proctitis, a form of UC. When the diagnosis of UC was made, he was administered a maintenance oral dose of 5-ASA, and no other immunosuppressive drugs (i.e., azathioprine and TNF inhibitors) were prescribed. Ten months before the onset of the sudden abdominal pain, a deep rectal ulcer was detected on endoscopy. An endoscopic examination revealed a grade 3 ulcer according to the Mayo endoscopic subscore (Fig. 1). Inflammation of the mucosa near the ulcer was slight and diagnosed as grade 1. On biopsy performed at the same time, there was no malignancy or cytomegalovirus (CMV) infection. The posology of 5-ASA was increased; he began taking a daily suppository of 5-ASA and Budesonide. Three months before the onset of the sudden abdominal pain, another endoscopic biopsy from the ulcer and pathological analysis were performed. Similar to the last time, the ulcer was diagnosed as grade 3, and there was no malignancy. Five days prior to the onset of the sudden abdominal pain, a daily dose of 40 mg of oral prednisolone was prescribed for the ulcer, as we clinically diagnosed that it did not respond to 5-ASA and Budesonide. Laboratory investigations revealed a slightly elevated C-reactive protein level, an elevated white blood cell count, no increase in tumor markers (carcinoembryonic antigen = 0.4 ng/mL, carbohydrate antigen 19–9 = 8.1 U/mL, carbohydrate antigen 125 = 14.4 U/mL), and CMV C 7-antigen. Preoperative investigation of soluble interleukin-2 receptor was not done. On the day of admission, the patient complained of severe lower abdominal pain. He had rebound tenderness with muscular defense. There was no fever, and laboratory tests showed non-elevated values for C-reactive protein (0.16 mg/L) and white blood cell count (7700/μL). Abdominal X-ray showed colon distension. Computed tomography (CT) of the abdomen and pelvis revealed intraperitoneal free air and an abnormal colon distension (Fig. 2a). The pelvic CT scan also suggested a perforation of the rectum but did not clearly reveal the presence of the lymphoma (Fig. 2b). These results indicated that the patient had a perforation rectal ulcer secondary to active UC. A subtotal colectomy with end ileostomy was performed. During the surgery, a fistula was discovered at the rectosigmoid region (Fig. 3). Pathological examination of the resected rectum revealed atypical lymphocyte infiltration in all of the layers of the rectal wall. Inflammation of the rectal mucosa in the non-perforated part was mild and evaluated as grades 1–2 according to Matt’s histological classification. There was no inflammation of the colonic mucosa (Fig. 4). Immunohistochemical staining was positive for the following: cluster of differentiation 20 (CD 20) markers, cluster of differentiation 10 (CD 10) markers, B-cell lymphoma 6 (BCL-6) proteins and multiple myeloma oncogene 1 (MUM-1)-stained atypical lymphocytes (Fig. 5). From these results, the pathological findings revealed that rectal perforation was related to DLBCL with UC. The stained specimen on immunohistochemistry was also positive for EBV (Fig. 6).

A postoperative fluorodeoxyglucose-positron emission tomography revealed an abnormal uptake in the postero-inferior area of the liver surface (S 7) and in the residual rectum margin. The standardized uptake value max (SUV max) of the liver region was 7.2, and the SUV max of the residual rectum was 11.1. Consequently, the patient was evaluated as having stage IV lymphoma according to the Lugano classification [14] and was treated with R-CHOP chemotherapy (rituximab, cyclophosphamide, doxorubicin, vincristine, and prednisolone) 2 months after the surgery. Six courses of chemotherapy were effective in correcting the abnormal uptake in the liver region without any complications. However, the abnormal uptake in the residual rectal wall slightly persisted after the six courses (SUV max = 3.6). Ileal pouch-anal anastomosis was performed 9 months after the primary surgery, and a histopathological examination of the residual rectum was done. There was no evidence of residual tumor, especially in the remnant rectal margin. The diverted ileostomy was closed, and he was disease-free. No recurrence was noted upon re-evaluation two years after his first surgery.

## Discussion and conclusions

The incidence of perforation in UC patients is rare because the inflammation in UC is usually limited to the submucosa. Patients usually take high doses of steroids or immunosuppressive drugs for the treatment in the active phase UC. CMV infection counteracts the use of immunosuppressive drugs and favors the development of toxic megacolon, which is a major cause of perforation in UC. The incidence rate of perforation in UC is reported to be around 3% [1].

However, the incidence rate of perforations in gastrointestinal lymphoma is approximately 9–22% and is higher than that in high-grade lymphoma (i.e., DLBCL) [2–4]. Primary gastrointestinal lymphoma may decrease the mechanical strength of the gastrointestinal wall and cause intestinal perforation [3, 15]. Some studies have found that perforation is a poor prognostic factor in patients with high-grade lymphomas like DLBCL [2–4, 16]. Vaidya reported that nearly half of the perforation events in gastrointestinal lymphoma occur at the time of diagnosis of gastrointestinal lymphoma suggesting that these perforations could not be avoided by prior interventions [3].Thus, it may be important to diagnose gastrointestinal lymphoma before it gets perforated, especially in UC patients.

The risk of gastrointestinal lymphoma is not higher in UC patients than in the general population [7]. Several previous studies have found that UC patients receiving thiopurines exhibit markedly increased relative risk of gastrointestinal lymphoma [9, 10]. Kandiel et al*.* reported an approximate fourfold increased risk of lymphoma in IBD patients treated with azathioprine [9]. Moreover, more than 90% of UC patients with gastrointestinal lymphoma receiving thiopurines complained of EBV infections [11]. Immunosuppressive therapy induces EBV infection and EBV-positive lymphomas (i.e., DLBCL). However, some studies have reported cases where DLBCL occurred in UC patients who were not administered immunosuppressive drugs [12, 13]. These cases suggest that DLBCL may occur regardless of whether immunosuppressive drugs are used or not.

In this case study, abnormal colon distension due to progression of the UC led to perforation of the DLBCL-induced ulcer. Immunosuppressive drugs were not administered, while steroids were administered only 5 days prior to the perforation. The incidence rate of perforation in patients with UC is low. However, whether the prevalence of gastrointestinal lymphoma in UC patients is high or not is still unclear, but the incidence rate of perforations in gastrointestinal lymphoma is relatively high. DLBCL may increase the frequency of perforation and is a poor prognostic risk factor for patients with UC. In this case, we performed two preoperative endoscopic biopsies of the ulcer, but we neither obtained a DLBCL-positive nor a CMV-infected specimen. DLBCL often causes ulcerations. Therefore, it is important to distinguish between a DLBCL ulcer and a UC ulcer by performing an endoscopic biopsy for choosing appropriate treatment for the ulcer. Hence, when a therapy-resistant colonic ulcer is detected in UC, biopsies from multiple points of the ulcer should be performed to confirm the presence of DLBCL to prevent perforation. In conclusion, gastrointestinal lymphomas such as DLBCL are rare in patients with UC, and bowel perforations induced by DLBCL ulcers in such patients are extremely rare. This case report emphasizes the importance of careful medical surveillance and repeated endoscopic biopsies in patients with UC.

## Data Availability

Not applicable.

## Abbreviations

5-ASA: 5-Aminosalicylic acid
CMV: Cytomegalovirus
DLBLCL: Diffuse large B-cell lymphoma
EBV: Epstein–Barr virus
IBD: Inflammatory bowel disease
MUM: Multiple myeloma oncogene
TNF: Tumor necrosis factor
UC: Ulcerative colitis
